# Scientific Items

**Published:** 1877-12-20

**Authors:** 


### Scientific Items.

EXPERIMENTS ON THE CIRCULATION IN THE BRAIN.—Some curious experiments have lately been made by a French physiologist upon the effects produced by increasing or dimishing the pressure of the blood in the brain. A rabbit, guinea pig, or dog is fixed to a horizontal plate, which is made to revolve at the rate of rather more than one turn in a second. If the head be towards the center of the apparatus, the centrifugal force drives the blood towards the tail end of the animal, producing a measure of bloodlessness in the brain. If the head be outermost, on the other hand, the rotation produces congestion of the nervous centres. In both cases, the animal, kept in a continual whirl for some time dies. It appears that death takes place much sooner in case of withdrawal of blood from the brain than in the case of brain congestion.

TELEPHONY.—The progress of telephony is something wonderful. The bell signal and the hand telephone have recently been invented. The bell signal requires no battery, and, indeed, the battery has been dispensed with in all the operations of Professor Bell's apparatus. By means of a magneto-electric device, the bells are struck at each end of the line by merely turning a wheel. The hand telephone is made of wood, and somewhat resembles a stethoscope, and is applied to the mouth or ear like the ordinary ear trumpet. Sounds are transmitted with great facility to great distances. It is thought that it will even be successfully applied to the Atlantic cable. There are now three hundred telephones operating in Boston, and they are being rapidly introduced into other places.

ELECTRIC PLANT.—In a German journal, a plant is described which emits electric shocks, when touched. It is said to affect the magnetic needle for a distance of several feet.

